# Research on the individualized short‐term training model of nurses in emergency isolation wards during the outbreak of COVID‐19

**DOI:** 10.1002/nop2.580

**Published:** 2020-08-04

**Authors:** Min Zhou, Fei Yuan, Xiaolong Zhao, Fanjie Xi, Xianxiu Wen, Li Zeng, Wenbo Zeng, Haiyan Wu, Hui Zeng, Ziyu Zhao

**Affiliations:** ^1^ Department of Urology Sichuan Provincial People's Hospital University of Electronic Science and Technology of China Sichuan China; ^2^ Department of Nursing Sichuan Provincial People's Hospital University of Electronic Science and Technology of China Sichuan China; ^3^ Department of Otolaryngology Head and Neck Sichuan Provincial People's Hospital University of Electronic Science and Technology of China Sichuan China; ^4^ Department of Infectious Disease Sichuan Provincial People's Hospital University of Electronic Science and Technology of China Sichuan China; ^5^ Department of Psychology College of Human and Health Sciences Swansea University Swansea UK

**Keywords:** COVID‐19, isolation, nurses, nursing, training

## Abstract

**Aim:**

To explore an effective personalized training model for nurses working in emergency isolation wards of COVID‐19 in a short period.

**Design:**

This study is a longitudinal study from 24 January 2020 to 28 February 2020.

**Methods:**

There are 71 nursing staff working in the emergency isolation wards of Sichuan Provincial People's Hospital that participated in this study. The questionnaires were conducted with Likert scale. The operation assessment teachers have received standardized training. The self‐rating anxiety scale (SAS) and self‐rating depression Scale (SDS) were applied to assess the mental state of nurses.

**Results:**

After short‐term training, these nurses can handle the emergency tasks in a timely manner. The pass rate of nurse theory and operation assessment is 100%. The 111 suspected patients admitted to the emergency isolation ward have been scientifically diagnosed and treated, the three confirmed patients have received appropriate treatment. No nurses have been infected.

**Conclusions:**

In this study, the personalized emergency training mode was feasible in the emergency isolation ward during the COVID‐19 epidemic, which rapidly improved the rescue ability of nurses and effectively avoid the occurrence of cross‐infection. This mode can provide a valuable reference for the emergency training of nurses in the future.

## 1 INTRODUCTION

COVID‐19 is an acute infectious pneumonia, which is a respiratory disease caused by a new coronavirus infection symptom, may combined with mild dry cough, fatigue, poor breathing, diarrhoea and other symptoms, catarrh and other symptoms such as runny nose and sputum are rare (National Health Commission of China). Respiratory droplets and contact transmission are the main transmission routes (National Health Commission of China). COVID‐19 broke out in Wuhan in December 2019, and then, the epidemic spread globally (WHO, 2020). The whole world should pay attention to how to deal with the outbreak (Kickbusch & Leung, 2020). To stop the spread of the epidemic as soon as possible, all of provinces and cities in China have taken strong measures. Although various industries have resumed production in China, the current international COVID‐19 epidemic is still grim. Under this circumstance, how to control the epidemic situation and prevent the recurrence of the COVID‐19 epidemic is worthy of attention (Wu, Guo, & Chao, 2020).

Through literature review, there are many studies on COVID‐19 in various countries; however, there are few reports about the training content of the nurses in the emergency isolation ward. Our training methods follows the model: training → assessment → feedback → evaluation → retraining → reassessment. Hopefully, this study could provide theoretical basis for training nurses under emergency assistance of COVID‐19 and we also hope to work with nursing colleagues around the world with an open attitude to save more patients.

### 1.1 Background

The Sichuan Provincial People's Hospital has temporarily established an emergency isolation ward during the outbreak of COVID‐19. To seek efficient nursing training mode under the epidemic situation and improve the nurses' knowledge reserve on emergency handling and control capabilities, a combination of on‐site training and online training was implemented to provide COVID‐19 related knowledge on nursing operation skills and hospital infections to the nursing team in a short term. The medical department, the infection control department and the nursing department trained the nursing staff through online social media (WeChat or OA system) and on‐site training. The nursing operations are mainly conducted through on‐site training. All nursing staff need to pass the assessment before they start to work in the emergency isolation ward. The electronic questionnaire was filled in after the nursing staff worked in the ward for 2 weeks to assess the nurses' need for training. Then, targeted training was conducted and the training results were evaluated by trainers.

## 2 MATERIALS AND METHODS

### 2.1 Reconstruction of emergency isolation wards

To control the spread of the epidemic, cut‐off the route of transmission and protect susceptible cases effectively, Sichuan Provincial People's Hospital reconstructed emergency isolation ward within three days. There are 32 open beds in this ward. By the end of 28 February (2020), a total of 111 suspected patients were treated in the emergency isolation ward, including three patients who were diagnosed with COVID‐19. They are mainly middle‐aged and elderly patients.

### 2.2 Preparation and management of the nursing team

During the preparation of the emergency isolation ward, the nursing department established the logistics team, professional team and management team immediately. The logistics team is responsible for participating in ward reconstruction and material preparation; the professional team is responsible for personnel training and supervision; and the management team is responsible for nursing staff management, including communication and coordination among various departments.

Firstly, the management team established a human resource database for emergency isolation ward. All nurses in the hospital are encouraged to apply online to the human resources database. Then, 71 nurses were selected by the nursing department from the human resources database. To avoid excessive fatigue, the selected nurses are divided into three echelons and work in turns on a flexible schedule. Nursing positions included clinical nurses, supervisors, trainers and head nurses.

### 2.3 Nursing staff training

The training process adopts training → assessment → feedback → evaluation → retraining → reassessment. A scenario drill is added in the first operation training section.

The training forms and content are shown in Table 1. The forms are including online training and on‐site training. The training contents include basic diagnosis, hospital infection, operation and psychological support.

**TABLE 1:** Training form and contents

Training form	Training contents		
Online training	Online video	**Ward layout** Guidelines on the use scope of common medical protective articles	National Health Commission of the People's Republic of China (2020a, 2020b)
	Online graphics	Graphic flow of putting on and taking off isolation clothes, graphic flow of washing hands, graphic flow of wearing masks, floor plan of ward	National Health Commission of the People's Republic of China (2020a, 2020b)
	Online text	Infection or infectious disease prevention measures, isolation and disinfection pollution knowledge; Disaster psychological knowledge Provisions for emergency treatment of public health events Disaster resource management and disease‐related nursing knowledge (US Centers for Disease Control and Prevention, 2019)	National Health Commission of the People's Republic of China (2020a, 2020b) US Centers for Disease Control and Prevention (2019)
On‐site training	On‐site theory	COVID‐19 diagnosis and treatment guidelines; Hospital infection guidelines	National Health Commission of the People's Republic of China (2020a, 2020b)
	On‐site operation	Diagnosis and treatment plan; operation of common medical protective equipment	National Health Commission of the People's Republic of China (2020a, 2020b)
	Live walkthrough	Ward layout, patient admission and workflow training	Sichuan Provincial People's Hospital COVID‐19 Work Guide
	On‐site psychological support	Mindfulness‐based stress reduction	Demarzo et al. (2015)

### 2.4 Disaster psychological knowledge training

Previous studies have already shown that nursing staff working in the emergency isolation wards might face tremendous psychological pressures (Oh et al., 2017). The anxiety self‐rating scale (SAS) and depression self‐rating scale (SDS) were implemented by head nurses to evaluate the psychological status of nursing staff.

Psychologists provide psychological support and guidance to medical staff based on individual circumstances, including online and on‐site psychological counselling (Zhou, 2020). In addition, experts from the psychological workshop were invited to perform mindfulness decompression. Psychological intervention based on mindfulness meditation has become an increasingly obvious part of the healthcare field (Demarzo, Cebolla, & Garcia‐Campayo, 2015).

### 2.5 Evaluation tool

Evaluation indicators include the following: nurses' grasp of training content, choice of training methods and improvement of psychological conditions before and after training. A self‐made online questionnaire “Nursing Staff Emergency Training Mode Approval Feedback Questionnaire” was used to access nursing staff's feedback on the emergency isolation ward's training mode. The questionnaire is divided into four parts and 20 items which mainly includes basic information, feedback on theory, operation training methods and content. In order to evaluate the degree of recognition, the training content evaluation is divided into three angles: text, graphics and video.

The knowledge level of the COVID‐19 is based on the National Health and Health Commission's diagnosis and treatment plan and the hospital's sense prevention and control requirements, including theory and operation. The “Nursing staff COVID‐19 emergency training theory assessment test questions” is prepared for online assessment. The operation technology is on‐site assessment. Then, comparing the pre‐training and post‐training, nursing theory and operation score are proportional to the degree of knowledge mastery.

The improvement of nursing staff's psychological construction level before and after the training adopts Zung's (1971) self‐rating anxiety scale (SRAS) and Zung's (1965) self‐rating depression scale (SRDS). Zung's SRAS and SRDS consist of 20 questions, each of which has answers in Likert scale format from levels 1–4. The original score is converted to 100 points and psychological evaluation of the nursing staff is performed. The higher the score, the greater the degree of anxiety and depression. Comparison was made before and after training.

### 2.6 Statistics

Statistical analysis was performed by SPSS13.0 software. Normal continuous variables were expressed as mean ± standard deviation, non‐normal continuous variables were expressed as median values (interquartile range) and categorical variables were expressed as percentages. The comparison between groups was based on whether they met the normal distribution using the Mann–Whitney U test or *t* test and the categorical variables used the chi‐square test or Fisher's exact test. Before and after the training, paired *t* test is mainly used; *p* < .05 was considered statistically significant.

### 2.7 Ethical considerations

The study was previously explained to the head nurse of the selected hospital, with official permission. The purpose of the study was explained to all study participants, and their informed consent was subsequently obtained. All answers are confidential and used for this study only.

## 3 RESULTS

Basic information of nursing staff. It is shown in Table 2 that all 71 nursing staff were female (35 nursing staff were 20–30 years old, accounting for 49.3%; 32 nursing staff were 31–40 years old, accounting for 45.07%; three were above 40 years old, accounting for 4.23%), 57 staff had an undergraduates degree, which accounted for 79.17%; 15 had a college degree or below, which accounted for 19.72%; and 41 staff had 5–10 years working experience (57.75%), 21 had 10 years and above experience (29.58%) and there were also nine staff whose working experience was under 5 years (12.68%). There were 36 internal nurses, accounting for 50.7%, 23 surgical nurses, accounting for 32.39% and six paediatric and obstetrics nurses, accounting for 8.45%. The mean age of nurses was 31.31 (*SD* 4.85), their working years ranged from 2 to 20 years.

**TABLE 2:** Basic situation of nursing staff (*n*, %)

Items	*n*	%
Gender		
Female	71	100
Age (years)		
20–30	35	49.3
31–40	32	45.07
41 above	3	4.23
Degree		
Bachelor degree	57	80.28
College degree and below	14	19.72
Clinical experience (year)		
≤5	9	12.68
5 < year ≤ 10	41	57.75
>10	21	29.58
Clinical department		
Internal medicine department	36	50.7
Surgery department	23	32.39
Paediatric department	6	8.45
Gynaecology and obstetrics	6	8.45

### 3.1 Nursing staff's degree of recognition of training method

Through the scale survey of nurses' degree of recognition of training methods, the results have shown that theoretical training, environmental and process training using online and offline combination (online + on‐site training) method is better Statistical significance (*p* = .042, *p* = .002); while the operation training adopts on‐site and on‐site + network training methods, the difference is not significant. (*p* = .081; Table 3).

**TABLE 3:** Nursing staff's recognition of training methods

Training form	On‐site (*n* = 71)	Online + On‐site (*n* = 71)	*p*
Theoretical training			
Very useful	64.5%	85%	.042
Useful	35.5%	15%	
Uncertain	0	0	
Useless	0	0	
Very useless	0	0	
Operation training			
Very useful	70%	86.8%	.081
Useful	30%	13.2%	
Uncertain	0	0	
Useless	0	0	
Very useless	0	0	
Drill training			
Very useful	58.6%	91.9%	.002
Useful	41.4%	8.1%	
Uncertain	0	0	
Useless	0	0	
Very useless	0	0	

### 3.2 Nursing staff's recognition of the training content

Nursing staff recognized and evaluated the training content in the form of questionnaires in terms of text, graphics and video. It is indicated that the operation training content with partial text and video was better (*p* = .042, *p* = .040). There is no difference in the training effect of the three types of network training content, theoretical training, environment and process training (Table 4).

**TABLE 4:** Nursing staff's recognition of training content

Training form	On‐site (*n* = 71)	Online + On‐site (*n* = 71)	*p*
Theoretical training			
Online text	80.48 ± 18.91	88.1 ± 16.67	.076
Online graphics	84.71 ± 14.72	89.95 ± 12.63	.111
Online video	88.45 ± 12.53	93.05 ± 10.64	.099
Operation training			
Online text	84.50 ± 14.65	91.34 ± 12.547	.042
Online graphics	84.17 ± 15.581	91.05 ± 13.41	.055
Online video	88.67 ± 13.16	94.29 ± 8.89	.040
Drill training			
Online text	84.90 ± 17.70	89.43 ± 13.475	.241
Online graphics	83.93 ± 15.33	90.59 ± 12.35	.055
Online video	89.21 ± 11.359	93.08 ± 9.181	.110

Compared with pre‐training and post‐training, the improvement of the COVID‐19 theory knowledge, operation skills and psychological conditions was significantly improved by paired *t* test, the mean value is >0 and the *p* value is <.01. The SAS score decreased after training, with statistical difference (*p* = .019). The difference in SDS before and after training was not statistically significant, with a *p*‐value equal to .306 (Table 5).

**TABLE 5:** Before and after training, the nursing staff improved the COVID‐19 theory knowledge, operation skills and psychological conditions

	Mean	*SD*	*p*
Theoretical results post‐training − Theoretical results pre‐training	20.268	14.922	.000
Results of putting on and taking off gowns after the first training − Results of putting on and taking off gowns before training	3.620	6.352	.000
Results of putting on and taking off gowns after the second training − Results of putting on and taking off gowns after the first training	14.352	12.996	.000
Results of wearing a mask after the first training − Results of wearing a mask before the training	2.676	4.662	.000
Results of wearing a mask after the second training − Results of wearing a mask after the first training	14.718	10.859	.000
The first round of hand washing results after training − The hand washing results before training	3.225	4.333	.000
The second round of hand washing results after training − The first round of hand washing results after training	14.225	10.288	.000
Post‐training SAS − Pre‐training SAS	−3.058	10.541	.019
Post‐training SDS − Pre‐training SDS	−1.986	16.212	.306

## 4 DISCUSSION

### 4.1 Nursing staff's understanding of COVID‐19 related theory

This study has shown that compared with the pre‐training, the difference between the theoretical and operational scores (wearing and taking off gowns, wearing masks, washing hands) after the training was significantly improved. The results reveal that the combination of online and on‐site training in an emergency can effectively improve the theoretical and operational level of nursing staff in a short period of time. In clinical research, the online + face‐to‐face hybrid approach is superior to two separate strategies (McCutcheon, Lohan, Traynor, & Martin, 2014; Terry, Terry, Moloney, & Bowtell, 2018), which is consistent with the results of this study.

There are several studies providing the basis of training cycles of nursing training programme. Some examples include a medical programme in Mumbai, India, mainly aimed to improve disaster nursing skills and knowledge, was set up for 7 days (Hilmi et al., 2011). Xu et al., (2020) reported that the training programme for COVID‐19 within 1–2 days, and the main content is nosocomial infection knowledge and operational skills. According to Gai et al. (2020) the nursing training for COVID‐19 lasted 3 days and the contents was like Xu's study.

Based on these evidences and the urgent situation under COVID‐19 Pandemic, the emergency isolation wards, emergency drills, theoretical and operational training were conducted for nursing staff in only three days. To ensure the safety of patients and nurses, a supervisor was set up to inspect and guide the nurses' work. Judging from the assessment results, the nurses adapted to the emergency well and no infections occurred. Nursing staff's acceptance of training content and methods in emergency situations.

In terms of the selection of training content, it is showed that most nursing staff need to receive training in COVID‐19 professional knowledge, hospital infection, surgery and psychology. One report from the US pointed out that the classroom/screen training content in emergency situations can be disaster resource management and disease‐related nursing knowledge (American Academy of Pediatrics, 2019a, 2019b; Centers for Disease Control and Prevention, 2019; National Library of Medicine, 2019; US Department of Health & Human Services, 2019a, 2019b), which has great clinical guiding significance for the selection of training content for this study.

In terms of the degree of recognition of the training content, the results of this study have shown that the order of online training scoring is as follows: online video, online text and online graphics. It illustrates that dynamic visualization training works best. And dynamic visualization training, such as animation and video, has been proved as a particularly effective teaching programme (Bernay & Betrancourt, 2016; Betrancourt & Tversky, 2000; Marcus, Cleary, Wong, & Ayres, 2013).

Regarding the selection of training methods, the results of the study have revealed that the on‐site training method is effective for the nursing staff to improve their ability in emergencies, and the combination of network and on‐site training methods is the best. Supported by research, the comparison between online and face‐to‐face training, well‐designed online training shows more advantages in terms of time efficiency and memory effect (Kalyuga, 2007; Kalyuga & Sweller, 2005), which is consistent with the results of this study. But it is less effective at changing behaviours (Aspegren, 1999; Mansouri & Lockyer, 2007) and face‐to‐face training seems to be more effective than online training. The reason for analysis may be related to the knowledge and skills provided by online training, and the on‐site training can improve the self‐confidence of nursing staff.

The psychological status of nursing staff before and after working in the emergency isolation ward.

The results of this study showed that the SAS score of nursing staff after standardized training was lower than before training (*p* < .05). It indicates that through training and psychological intervention (mindfulness, group) related to the COVID‐19, the nurses in the emergency ward can be guided to scientifically treat the infection and control of the COVID‐19, thereby reducing the level of anxiety. The SDS level did not change much before and after, one of the possible reasons could be the short‐term trainees did not reach the level of depression. However, although the training involves psychological aspect, the need for psychological support is repeatedly mentioned in the questionnaire, indicating that when the medical staff first contacted the patient at the beginning of the outbreak and faced a large number of patients (Suwantarat & Apisarnthanarack, 2015), the nurse's level of occupational stress is increasing. (Wheeler, 1997).

### 4.2 Limitations

Due to the short‐term emergency training and the high risk of infection, it is a huge challenge work for nursing staff. The survey shows that psychological problems are repeatedly mentioned, indicating that nurses have a greater need for psychological support, which seemingly suggests that future psychological support for front‐line nurses needs to be strengthened in many ways (Zhou, 2020).

Selecting the nursing staff of the emergency ward of a hospital may have certain geographical restrictions, or the sample size may not be large enough. More samples from different regions can be researched in the future.

The theory of planned behaviour holds that past experience is one of the determinants of a person's beliefs (Ajzen, 1991; Oh et al., 2017). The planned selection of experienced nursing staff to participate in the rescue incident is more conducive to the rescue work. This training did not involve the problems of personnel's previous rescue experience. In future emergency rescue work, it is preferable to choose personnel with rescue experience in the human resources database.

## 5 CONCLUSION

Emergency training of nursing staff is crucial on preventing the spread of the COVID‐19 epidemic effectively and ensuring the operation of emergency isolation ward orderly. The training content of this study is based on the COVID‐19 Theory operating materials of the Chinese Health Commission and the US guidelines for disease prevention. The training form is a combination of online and offline. In order to form the best training content and provide an optimized training model for the next epidemic prevention and control, the effectiveness of the training form was analysed. At the same time, this study pays particular attention to the psychological problems of nursing staff. Carrying out the prevention and intervention of psychological problems to the nursing staff in a timely manner will ensure the staff positively faces the epidemic situation. In short, the value of nursing staff in the prevention and control of the COVID‐19 epidemic cannot be replaced. How to ensure the safety of nursing staff and patients through training is a significant issue that worth to be discussed.

## AUTHOR CONTRIBUTIONS

Fei Yuan, Min Zhou and Xiaolong Zhao: Access to all of the data in the study and responsible for data integrity and accuracy of data analysis. Xiaolong Zhao, Fei Yuan, Fanjie Xi, Xianxiu Wen and Ziyu Zhao: Study design. Fei Yuan, Fanjie Xi, Hui Zeng, Li Zeng, Haiyan Wu and Wenbo Zeng: Data collection. Xiaolong Zhao, Min Zhou and Fei Yuan: Statistical analysis. Min Zhou, Fei Yuan, Xiaolong Zhao and Ziyu Zhao: Manuscript draft.
